# Optimising low-energy defibrillation in 2D cardiac tissue with a genetic algorithm

**DOI:** 10.3389/fnetp.2023.1172454

**Published:** 2023-07-24

**Authors:** Marcel Aron, Thomas Lilienkamp, Stefan Luther, Ulrich Parlitz

**Affiliations:** ^1^ Institute of Pharmacology and Toxicology, University Medical Center Göttingen, Göttingen, Germany; ^2^ Max Planck Institute for Dynamics and Self-Organization, Göttingen, Germany; ^3^ Institute for the Dynamics of Complex Systems, Georg-August-Universität Göttingen, Göttingen, Germany; ^4^ German Center for Cardiovascular Research (DZHK), Partner Site Göttingen, Göttingen, Germany; ^5^ Computational Physics for Life Science, Nuremberg Institute of Technology Georg Simon Ohm, Nuremberg, Germany

**Keywords:** cardiac arrhythmias, ventricular fibrillation, low-energy defibrillation, chaos control, excitable media

## Abstract

Sequences of low-energy electrical pulses can effectively terminate ventricular fibrillation (VF) and avoid the side effects of conventional high-energy electrical defibrillation shocks, including tissue damage, traumatic pain, and worsening of prognosis. However, the systematic optimisation of sequences of low-energy pulses remains a major challenge. Using 2D simulations of homogeneous cardiac tissue and a genetic algorithm, we demonstrate the optimisation of sequences with non-uniform pulse energies and time intervals between consecutive pulses for efficient VF termination. We further identify model-dependent reductions of total pacing energy ranging from *∼*4% to *∼*80% compared to reference adaptive-deceleration pacing (ADP) protocols of equal success rate (100%).

## 1 Introduction

Sudden cardiac death (SCD) accounts for an estimated 15% of worldwide mortality (Mehra, 2007). SCD is caused by malignant tachyarrhythmias that are associated with spatiotemporal chaos in the heart (Winfree, 1989; Davidenko et al., 1992; Gray et al., 1998; Witkowski et al., 1998; Cherry and Fenton, 2008; Christoph et al., 2018). For a lack of a better strategy, high-energy electric shocks are used to terminate electrical activation in the entire heart. However, high-energy shocks have significant side-effects, including tissue damage (Xie et al., 1997; Tereshchenko et al., 2009), traumatic pain (Godemann et al., 2004; Marcus et al., 2011), and worsening of prognosis (Mackenzie, 2004; Poole et al., 2008; Moss et al., 2012), indicating a significant medical need for improvement (Babbs et al., 1980).

To address this need, pacing methods have been developed that aim to replace the single high-energy shock with a sequence of low-energy pulses. In general, these control algorithms differ in detail in the generation of the pulse sequences and are either open or closed-loop. Low-energy anti-fibrillation pacing (LEAP) uses a sequence of electrical pulses of constant amplitude, duration, and period to terminate atrial and ventricular fibrillation (Fenton et al., 2009; Luther et al., 2011; Buran et al., 2022). Using this approach in *in-vivo* experiments, a pulse energy reduction of 80%–90% compared to the conventional single shock has been achieved. However, several studies showed that the performance of pacing protocols with equidistant pulses depends very sensitively on the choice of the pacing interval (Buran et al., 2017; DeTal and Fenton, 2021; Lilienkamp et al., 2022a; Buran et al., 2023). As another example, multi-stage pacing developed by the Efimov lab employs groups of pulses with constant amplitude and timing (Janardhan et al., 2014).

Several numerical and experimental studies have investigated mechanisms related to arrhythmia control and defibrillation (Trayanova et al., 2011; Rantner et al., 2013), including the formation of virtual electrodes (Efimov and Ripplinger, 2006), interaction of electric fields with cardiac tissue and curved boundaries (Pumir and Krinsky, 1999; Bittihn et al., 2012), the unpinning of rotational waves (Pumir et al., 2007; Bittihn et al., 2008; Bittihn et al., 2010; Shajahan et al., 2016; Boccia et al., 2017a; Boccia et al., 2017b; tom Wörden et al., 2019), the effect of pacing frequency (Hornung et al., 2017), the electric field geometry (Otani et al., 2019) and electrode placing (Trayanova and Chang, 2016).

Experimentally, optogenetically modified cardiac cells offer new ways for implementing and testing alternative approaches for defibrillation (Bruegmann et al., 2016; Hussaini et al., 2021). Progress has also been been made combining experimental data with numerical modelling, for example, for studying spatiotemporal alternans patterns of cardiac excitation (Loppini et al., 2022). Recent numerical studies have shown that pulse timing is crucial for termination success (Steyer et al., 2023). Furthermore, chaotic cardiac arrhythmias may exhibit self-termination (Rappel et al., 2022), a phenomenon also seen with chaotic transients in generic excitable media (Lilienkamp et al., 2017; Lilienkamp and Parlitz, 2018; Aron et al., 2019). The fact that transient chaos in excitable media can in principle be terminated by very few local perturbations (Lilienkamp and Parlitz, 2020) provides additional evidence that low-energy defibrillation is in principle possible. And in fact, recent studies show progress in the quest of finding better defibrillation methods, for example, exploiting targeted manipulation of spiral waves (DeTal et al., 2022), non-monotonous dose-response curves (Lilienkamp et al., 2022a) and decelerated pulse trains (Lilienkamp et al., 2022b).

In particular, the progress made with non-equidistant pulse sequences (Lilienkamp et al., 2022b) raises the question of further increasing termination efficiency when more complex sequences of (local) perturbations are used. However, the systematic experimental optimisation of such arrhythmia control algorithms with a large parameter space is under practical and ethical constraints not feasible. Therefore, in contrast to the above mechanistic, hypothesis-driven studies, we formulate the control of spatio-temporal dynamics in this study as an optimisation problem and use a *genetic algorithm* (GA) to cope with the vast landscape of possible pacing protocols spanning all combinations of pulse counts, amplitudes, and intervals between pulses.

Following natural evolution, the genetic algorithm mutates and combines pacing sequences and evaluates their “fitness” in terms of (high) termination probability and (low) pulse amplitudes (or energies). Because the goal of this study is to demonstrate the feasibility of GA optimisation of low-energy defibrillation in a proof of principle, we use simple phenomenological models of cardiac tissue in a homogeneous, isotropic 2D medium to avoid excessive computational burden that would result from more realistic modeling. This requires an appropriate selection of cardiac-tissue models as a foundation for simulations of two-dimensional sheets of homogeneous cardiac tissue, as well as a simplified translation of real-life defibrillation into something a computer can parse in reasonable amounts of time. This simulation framework will be introduced in the following section.

## 2 Simulating ventricular fibrillation

In this section, we present the cardiac-tissue model(s) and numerical integration required to study and evaluate different pacing sequences.

### 2.1 Cardiac-tissue models

Cardiac-tissue models describe the electrophysiological dynamics of the heart. The heart contracts (i.e., pumps) in response to electrical excitation-waves propagating through its muscle tissue in a coordinated fashion. Such waves originate in the sinoatrial node in regular intervals to drive the heartbeat. Because cardiac tissue constitutes an excitable medium, orderly propagation is generally ensured: Each cell only responds to (and propagates) incoming signals above a certain activation threshold and then enters a temporary post-excitation refractory period where further propagation is greatly impeded. While the former prevents (weak) abnormal activation, the latter ensures the unidirectional movement of the signal for the coordinated contraction of each heart chamber. Both mechanisms are a result of ion channels permeating cardiac muscle cells, which open and close in a predetermined fashion upon activation. They allow for the migration of various ion species to and from the cell’s interior, changing the net voltage across the cell membrane in the process before eventually returning it to its resting state.

We limited ourselves to *phenomenological* cardiac-tissue models for the pacing simulations for reasons of efficiency. Such models attempt to establish an abstract, high-level description of the complex physics involved in cardiac electrophysiology at manageable levels of mathematical complexity. This means they naturally lend themselves to large-scale computer simulations and experiments as they are usually both simpler to implement and less demanding of computational resources. These are in contrast to physiological models, which incorporate some (or more) of the underlying physiological details contributing to the signal-propagation dynamics in cardiac tissue. The incorporation of such details usually incurs significant additional computational cost, and we thus restricted our study to phenomenological models to reduce overall simulation time.

A generic cardiac-tissue model is given by a reaction-diffusion equation for the transmembrane potential *u*, the net voltage across a local aggregate (continuum) of cell membranes:

$$\partial_{t}u=\nabla \cdot \left(D\;\nabla u\right)-\frac{I_{tot}}{C_{m}}.$$

A local *net current* *I*_tot_ makes up the reaction term which may amplify or counteract the ion-diffusion process to neighbouring cells as governed by the *diffusion tensor* *D*. The net current consists of a model-dependent number of contributing currents modulated by a number of ion-channel gates whose permeabilities are represented by a set of dimensionless *gating variables*. *C*_m_ is the cell-membrane capacitance. Lastly, the *u* is subject to a no-flux Neumann boundary condition where $\nabla u\cdot \hat{n}=0$ (with $\hat{n}$ as the boundary normal) holds everywhere on the physical boundary of the simulated tissue.

Throughout this paper, we generally refer to chaotic states of cardiac-tissue models as *fibrillation episodes* in reference to the actual medical condition of (ventricular) fibrillation which also features chaotic electrophysiological dynamics. The simulated control-methods used, which aim to terminate such chaotic states, are analogously called *defibrillation* methods in this context. However, it should be understood that two-dimensional simulations of homogeneous cardiac tissue and their control by local current injection imitating virtual electrodes represent a substantial simplification of the real heart. Accordingly, great care must be taken in generalising any observations and findings to the broader medical context.

#### 2.1.1 The Fenton-Karma model

**FIGURE 1:**
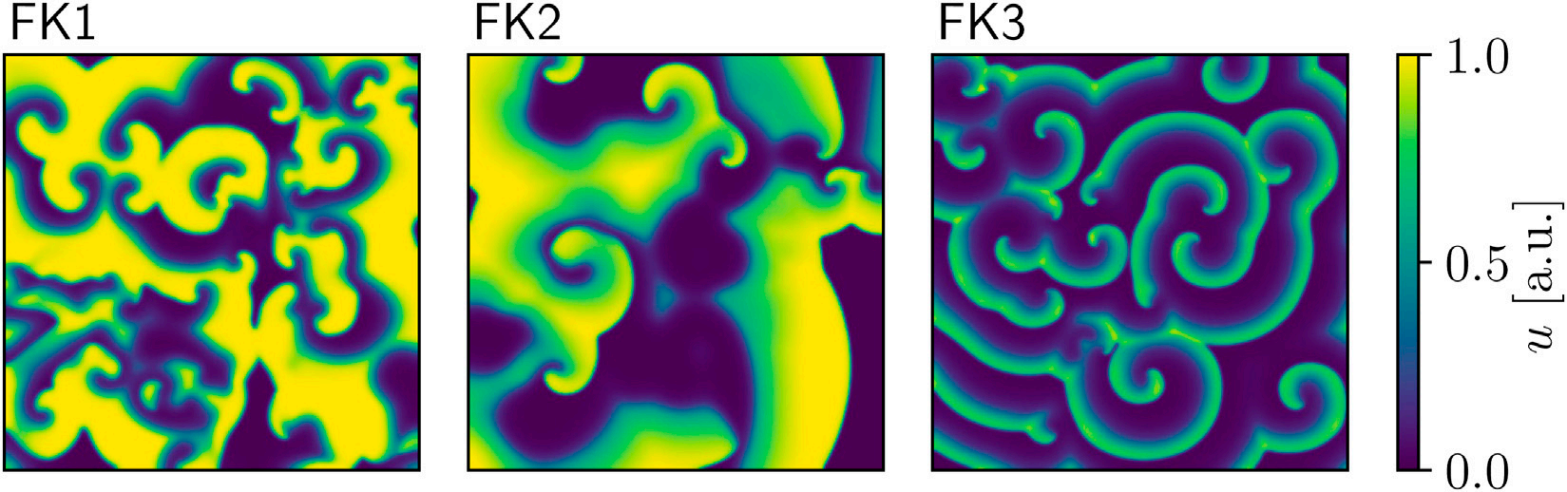
Snapshots of voltage spiral-waves for each of the three FK parameter sets.

Our first model of choice was the *Fenton-Karma* (FK) *model* (Fenton and Karma (1998); Figure 1) for human ventricular tissue. It offers great versatility in its behaviour through its various high-level parameters (Fenton et al., 2002) and it can in fact be fine-tuned to approximate the dynamics of more complex (e.g., physiological) models as needed. Employing multiple FK parameter sets (see Tables 1, 2) enabled us to test any potential pacing-protocol improvements for one set for reproducibility with others at little extra computational cost.

**TABLE 1:** Parameter values and dimensions for the three FK models.

Param.	Unit	FK1	FK2	FK3
*D*	mm^2^/ms	0.20	0.20	0.20
*C*_m_	ms	1	1	1
${\tau_{v}}^{+}$	ms	13.030	3.330	3.330
${\tau_{v1}}^{-}$	ms	19.6	9.0	19.6
${\tau_{v2}}^{-}$	ms	1250	8	1250
${\tau_{w}}^{+}$	ms	800	250	870
${\tau_{w}}^{-}$	ms	40	60	41
*τ*_d_	a.u.	0.450	0.395	0.250
*τ*_0_	1	12.5	9.0	12.5
*τ*_r_	a.u.^−1^	33.25	33.33	33.33
*τ*_si_	a.u.^−1^	29	29	29
*K*	a.u.^−1^	10	15	10
$u_{c}^{si}$	a.u.	0.85	0.50	0.85
*u*_c_	a.u.	0.13	0.13	0.13
*u*_*v*_	a.u.	0.04	0.04	0.04

**TABLE 2:** Overview of the cardiac-tissue models used in our computer simulations. The dominant periods were estimated through Fast Fourier Transforms applied to time series produced by these models.

Model	Param. set	***T***_**dom**_ **[ms]**	Reference
FK	1	130	Table 1, column 8 in Fenton et al. (2002)
	2	157	Table 1, column 6 in Fenton et al. (2002)
	3	68	Table 1, column 3 in Fenton et al. (2002)
BOCF	1	337	Table 1, column “TNNP” in Bueno-Orovio et al. (2008)

The model itself features three dimensionless state variables: the transmembrane potential (*u*) and two gating variables (*v*, *w*). The gating variables govern two of the three transmembrane currents found in this model’s net-current equation:

$$\begin{array}{ll} I_{tot} & =I_{\mathrm{fi}}\left(u,v\right)+I_{so}\left(u\right)+I_{si}\left(u,w\right)+I_{stim}\left(\vec{x},t\right),\\ \partial_{t}v & =\frac{1}{{\tau_{v}}^{-}}H\left(u_{c}-u\right)\left(1-v\right)-\frac{1}{{\tau_{v}}^{+}}H\left(u-u_{c}\right)\;v,\\ \partial_{t}w & =\frac{1}{{\tau_{w}}^{-}}H\left(u_{c}-u\right)\left(1-w\right)-\frac{1}{{\tau_{w}}^{+}}H\left(u-u_{c}\right)\;w, \end{array}$$

where *H* denotes the standard Heaviside step-function and *I*_stim_ is an external stimulation (e.g., pacing-induced) current. $\tau_{v}^{-}\left(u\right)=\Theta \left(u-u_{v}\right)\tau_{v1}^{-}+\Theta \left(u_{v}-u\right)\tau_{v2}^{-}$ is dependent on *u*, making it a *time constant* in need of constant re-evaluation during the integration process. The diffusion tensor *D* was assumed isotropic with a scalar value of *D* = 0.20 mm^2^/ms and the capacitance set to *C*_*m*_ = 1 ms. The FK model’s transmembrane voltage *u* is normalised such that its resting value lies at *u* = 0.0 a.u. for all its parameter sets, making comparisons between them straightforward.

#### 2.1.2 The Bueno-Orovio-Cherry-Fenton model

**FIGURE 2:**
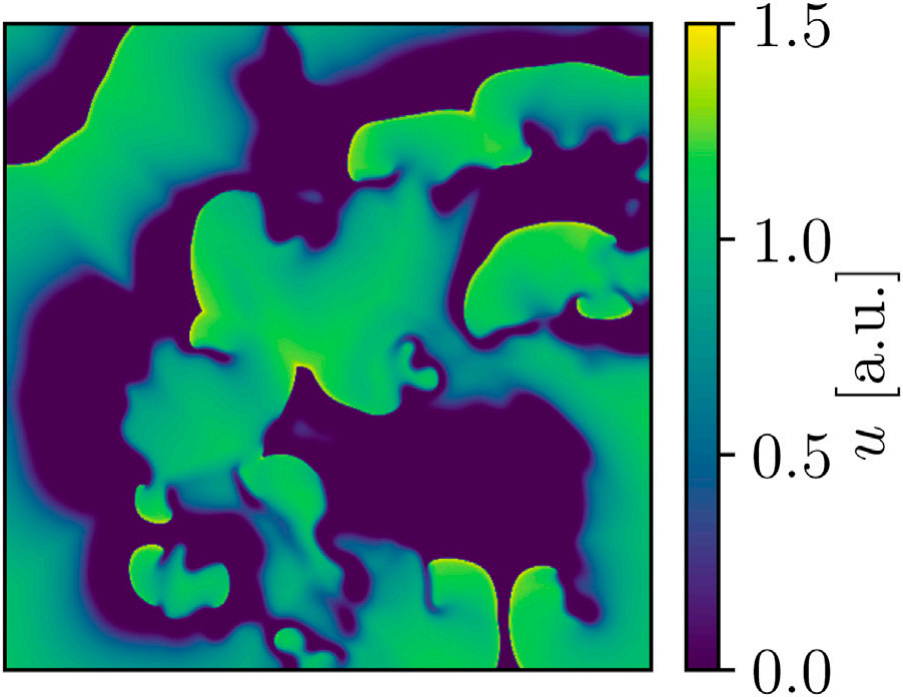
Snapshot of BOCF voltage dynamics. Area-wise, this domain is four times larger than the one used for the FK models, and the peak voltage of the model’s action potential is also notably higher at approx. 1.5 a.u. in comparison.

The *Bueno-Orovio-Cherry-Fenton* (BOCF) *model* (Bueno-Orovio et al. (2008); Figure 2) was our second cardiac-tissue model of choice, which also describes the dynamics in human ventricular tissue. Like the FK model, the BOCF model is also capable of approximating other models on a qualitative level given proper parameters. It features four currents and three gating variables:

$$\begin{array}{ll} I_{tot} & =I_{\mathrm{fi}}\left(u,v\right)+I_{so}\left(u\right)+I_{si}\left(u,w,s\right)+I_{stim}\left(\vec{x},t\right),\\ \partial_{t}v & =\frac{1}{{\tau_{v}}^{-}}\left[1-H\left(u-\theta_{v}\right)\right]\left(v_{\infty }-v\right)-\frac{1}{{\tau_{v}}^{+}}H\left(u-\theta_{v}\right)\;v,\\ \partial_{t}w & =\frac{1}{{\tau_{w}}^{-}}\left[1-H\left(u-\theta_{w}\right)\right]\left(w_{\infty }-w\right)-\frac{1}{{\tau_{w}}^{+}}H\left(u-\theta_{w}\right)\;w,\\ \partial_{t}s & =\frac{1}{\tau_{s}}\left[\frac{1}{2}\left(1+\right.\tanh\left(k_{s}\left(u-u_{s}\right)\right)-s\right]. \end{array}$$

The model has seven time constants, mostly in its *τ* parameters. Its resting potential is also normalised to 0.0 a.u. and the membrane capacitance set to unity (ms). We similarly assumed an isotropic *D* = 0.30 mm^2^/ms as well. We used a single parameter set (see Tables 2, 3) designed to emulate the behaviour of a much more complex physiological model in our simulations.

**TABLE 3:** Parameter values and dimensions for the BOCF model.

Param.	Unit	BOCF
*D*	mm^2^/ms	0.30
*C*_m_	ms	1
*θ*_*v*_	a.u.	0.3
${\theta_{v}}^{-}$	a.u.	0.015
*θ*_*w*_	a.u.	0.015
*θ*_o_	a.u.	0.006
${\tau_{v}}^{+}$	Ms	1.4506
${\tau_{v1}}^{-}$	ms	60
${\tau_{v2}}^{-}$	ms	1150
${\tau_{w}}^{+}$	ms	280
${\tau_{w1}}^{-}$	ms	70
${\tau_{w2}}^{-}$	ms	20
*τ*_fi_	1	0.11
*τ*_o1_	1	6
*τ*_o2_	1	6
*τ*_so1_	1	43
*τ*_so2_	1	0.2
*τ*_s1_	ms	2.7342
*τ*_s2_	ms	3
*τ*_si_	1	2.8723
*τ*_*w*__*∞*_	a.u.	0.07
${w_{\infty }}^{*}$	1	0.94
${k_{w}}^{-}$	a.u.^−1^	65
*k*_so_	a.u.^−1^	2
*k*_*s*_	a.u.^−1^	2.0994
*u*_o_	a.u.	0
*u*_u_	a.u.	1.58
${u_{w}}^{-}$	a.u.	0.03
*u*_so_	a.u.	0.65
*u*_*s*_	a.u.	0.9087

### 2.2 Numerical implementation

We limited simulations to square, isotropic 2D tissue sheets for speed and ease of implementation. We chose domain sizes spanning 256^2^ and 512^2^ pixels for the FK and BOCF model(s), respectively. Both had the same discretisation parameters of Δ*t* = 0.1 ms for the time step and $h\overset{def.}{=}\Delta x=\Delta y=1.0\;\;mm$ for the spatial (grid) resolution.

We discretised voltage variables with a *Forward in Time, Central in Space* (FTCS) scheme, which is an explicit first-order method in both time and space (Li et al., 2018). For the Laplacian, we used a 9-stencil approximation (Lynch, 1992) in place of the 5-stencil at non-vertex voltage-grid locations (*i*, *j*) to ensure a more radially independent discretisation error for proper spiral-wave propagation.

The separate treatment of vertices in the computation of the Laplacian is owed to our reliance on the “ghost point” method for the enforcement of the no-flux boundary condition on the voltage grid *u* (Li et al., 2018). This method extends the grid representing *u* by 2 pixels along each axis (one per side for a rectangular domain), creating a one-pixel boundary around it. Each such pixel is linked to another specific pixel of the interior grid (either one row or column apart) such that they both always hold the same value. Applying FTCS and Laplace discretisations on the main part of this extended grid then enforces the boundary condition automatically. The 5-stencil approximation is thus needed to compute the Laplacian on any of the interior grid’s four vertices to avoid referencing the undefined values in any of the extension’s four corners.

## 3 Modelling and evaluating pacing protocols

2D simulations of heterogeneous cardiac tissue by themselves lack the means to reproduce the defibrillation mechanisms of real hearts in an emergent fashion. We therefore had to model and introduce the qualitative properties of these mechanisms to our simulations manually to enable the simulation of pacing protocols. We focused on the mode of operation of low-energy pacing in particular for the modelling process.

### 3.1 Low-energy pacing and virtual electrodes

Despite advances in defibrillator design, the currents and energies involved still carry substantial risk to the patient’s long-term health and may cause internal tissue damage or even long-term changes in a given patient’s heart rhythm. This is the principal motivation behind contemporary *low-energy defibrillation* research (Pumir et al., 2007), which seeks to discover and study novel, potentially less intrusive pacing approaches such as low-energy anti-fibrillation pacing (LEAP) (Luther et al., 2011). LEAP replaces the singular, potent mono- or biphasic shocks with a sequence of temporally equidistant, uniformly low-amplitude ones to lower the risk of harmful side effects to the patient.

Low-energy pacing cannot rely on overwhelming the fibrillating signal in the heart by forcefully activating most muscle tissue; it exploits the numerous small conductivity heterogeneities (e.g., blood vessels, scar or fatty tissue) permeating cardiac tissue to perturb the electric activity in the heart instead. Such a heterogeneity can function as a local signal wave-front emitter by acting as an anode-cathode pair (a *virtual electrode*) when exposed to an electric field of sufficient strength and proper orientation. Such locally emitted wave fronts can then interact with the fibrillating signal in the heart by blocking its propagation through the refactory period they induce at various locations in the affected tissue, ideally putting an end to the fibrillation episode.

A virtual electrode’s activation threshold and response depend on its geometry and orientation relative to the external electric field (Bittihn et al., 2008; Bittihn et al., 2012), where higher field strengths can generally be used to “recruit” more of these electrodes for defibrillation purposes. Low-energy defibrillation methods capitalise on low-threshold virtual electrodes which can trigger in response to weaker fields, potentially disrupting a fibrillation episode through a few periodic (in place of just a single potent one as used in traditional defibrillation) activations and subsequent emissions at minimal overall risk to the patient. While low-energy defibrillation methods such as LEAP have seen success in laboratory settings, they are still pending adoption in the medical context until further clinical trials have fully established their overall viability.

### 3.2 Our simplified pacing model

A pacing protocol comprises a sequence of *pacing amplitudes* and *inter-pulse intervals*. We preemptively reduced the dimensionality of the protocol-optimisation space by limiting the search to protocols of a uniform pulse length of 2.0 ms throughout all our simulations. Similarly, we further only considered monophasic protocols of positive amplitude(s) for our study. Under these constraints, a monophasic *N*-pulse protocol therefore featured *N* amplitudes and *N* − 1 inter-pulse intervals for a total of 2 *N* − 1 parameters. We conducted our investigation with 5-pulse protocols first, which still left us with 9 protocol parameters to optimise.

In a defibrillation attempt based on a given protocol, we would first apply it through randomly distributed *injection sites* modelling the “recruitment” of local virtual electrodes for pacing purposes as utilised in low-energy defibrillation approaches. We then observed the system for a short amount of time and employed a simple binary criterion based on the resulting *peak transmembrane voltage* across the tissue domain to check for successful defibrillation afterwards: If max(*u*(*t*)) < *u*_crit._ = 5 × 10^−2^ a.u. holds across the entire tissue domain at time *t*, then no further action potentials can occur and the system is rapidly approaching its resting state. We used a global value for *u*_crit._ as all our cardiac-tissue models of choice featured normalised voltages variables.

**FIGURE 3:**
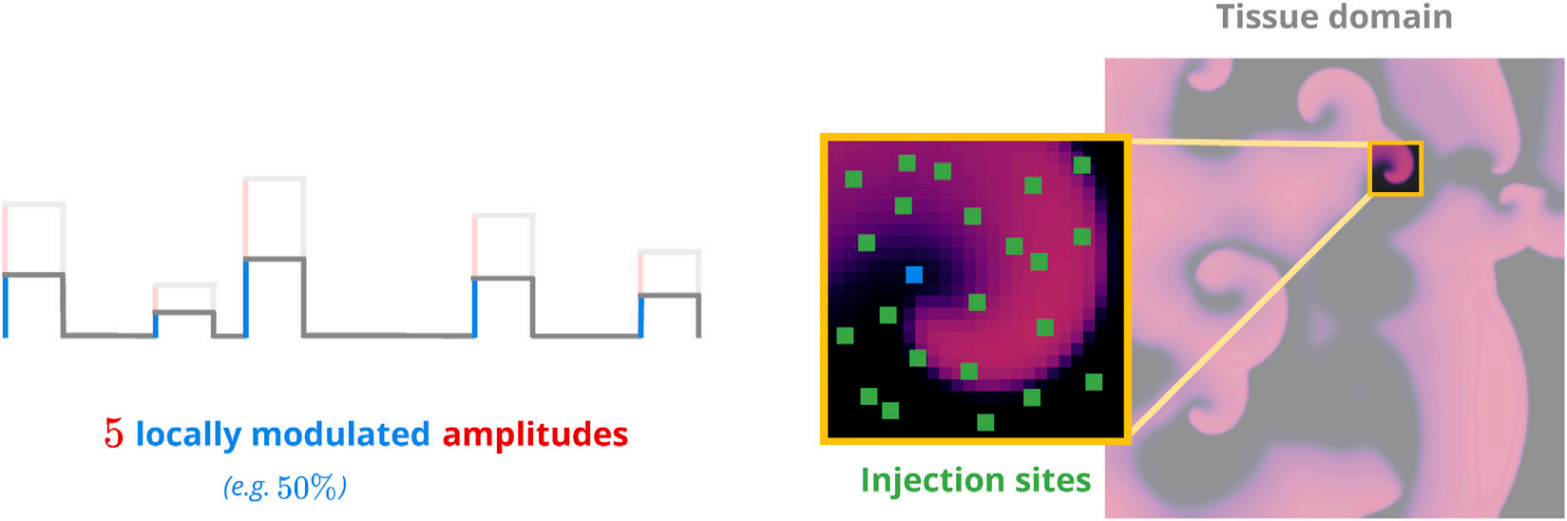
Sketch of the pacing model. Non-overlapping injection sites (green) are distributed across the voltage domain (right panel). Each site has a random efficacy coefficient to modulate the amplitude(s) of the pacing protocol to be applied (left panel). This emulates the effects of different heterogeneity geometries on a local level.

We applied pacing protocols numerically through local, time-dependent changes in a given cardiac-tissue model’s stimulation current $I_{stim}\left(\vec{x},t\right)$ as specified by protocol parameters. The individual areas of stimulation were chosen by randomly distributing non-overlapping injection sites spanning 3 × 3 pixels each (Figure 3) throughout the tissue domain. Each injection site was further associated with a uniformly drawn *modulation coefficient* between 0 and 1 which defined the (relative) local pacing efficacy at a given site to account for the effects of non-uniform virtual-electrode geometries and orientations in live tissue. We chose an injection-site coverage of *∼*28% as an arbitrary starting point to account for the number of virtual electrodes involved, which translated to 2,000 and 8,000 injection sites for the FK and BOCF model(s), respectively. Once generated, both the injection-site locations and their respective modulation coefficients remained constant throughout the application of a given protocol.

The length of the post-pacing observation period was model-dependent at a value of 5 × *T*_dom_, where *T*_dom_ denotes the *dominant period of oscillation* for a given model. While potentially chaotic systems like the cardiac-tissue models may not conform to a single period of oscillation, it is still possible to estimate a dominant (i.e., most prevalent in terms of its absolute Fourier-spectrum contribution) one by means of *fast Fourier transforms* (Schwaderlapp et al., 2017). This period can be understood as something resembling a mean period of oscillation for the wave fronts in the chaotic dynamics. As we generally want defibrillation to occur in a reasonable time frame, we settled on this 5 × *T*_dom_-interval so as to enforce this criterion.

To estimate the models’ dominant periods (see Table 2), we integrated and observed one initial condition per model over an interval of 10 s at 120 samples per second, yielding one time-series of 1200 data points per pixel of the discretised voltage domain. The separation of the domain into individual time-series in this step can be justified with the spatiotemporally chaotic nature of the dynamics, where spatial (and temporal) correlations decay over short distances (or time intervals). We then applied a Fast Fourier Transform to the resulting time series, computed their highest-contributing frequencies, and subsequently averaged them to estimate “the” dominant frequency (and therefore period) of the given model.

We detected successful defibrillation following the post-pacing observation period by checking whether max(*u*) < 0.05 a.u. held for the resulting voltage grid. If it did, the system would be incapable of creating further wave fronts on its own and could therefore be considered defibrillated. We determined this threshold manually and applied it uniformly to all our selected cardiac-tissue models because of their common voltage ranges.

### 3.3 Defining protocol-performance metrics

**FIGURE 4:**
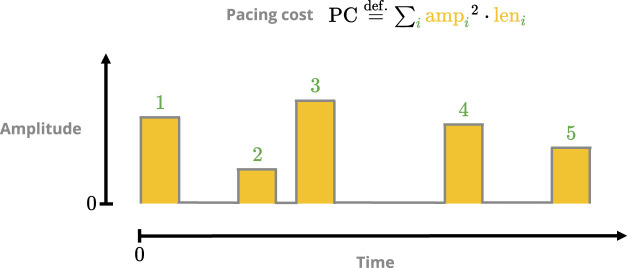
Sketch of the pacing-cost metric. The square of each pulse amplitude is integrated over its respective duration, and the sum over all pulses then yields the total cost. The squaring is done to penalise protocols with overly potent singular pulses.

We defined two performance metrics for pacing protocols: *pacing cost* (PC) and *termination ratio* (TR). PC measures the theoretical cost in applying a protocol based on its pulse amplitudes (analogous to energy; Figure 4), with lower values being preferred. A given protocol’s TR is an estimate of its efficacy in terminating (i.e., defibrillating) simulated episodes of fibrillation; it ranges in value from 0 (completely ineffective) to 1 (peak efficacy).

While PC can be computed at negligible cost for a given protocol, its TR value needs to be estimated by sampling the protocol’s defibrillation efficacy through computationally expensive simulations. We conducted the sampling process through a series of defibrillation simulations involving a predetermined set of 10 model-dependent *initial conditions* (ICs) representing fibrillation states. This process was repeated a predetermined number of times to account for the influence of different injection-site distributions (e.g., 5 distributions each, amounting to 5 × 10 samples per protocol). The TR value was then set to the resulting success (i.e., defibrillation) ratio among these samples. Two videos demonstrating a successful and failed termination attempt, respectively, are provided as Supplementary Material.

It should be noted that fibrillation episodes in our cardiac-tissue models of choice are of an inherently transient nature (Strain and Greenside, 1998; Lilienkamp et al., 2017; Lilienkamp and Parlitz, 2018; Aron et al., 2019) such that any chaotic trajectory ultimately converges to a steady (i.e., “defibrillated”) state of its own, and this also applies to the general post-pacing (i.e., perturbation) state of such an episode. As the mean *transient time* for this transition grows exponentially with the physical domain size to which the dynamics are confined, our choice of sufficiently short post-pacing observation periods kept potential biases in our TR estimates through such “self-terminated” fibrillation episodes to a minimum.

### 3.4 Choosing reference protocols

We needed reference points to gauge prospective pacing-optimisation results. To that end, we consulted the *adaptive (spectrum-guided) deceleration pacing (ADP)* scheme (Lilienkamp et al., 2022b) to derive an “optimal” set of reference protocols.

ADP protocols feature pulses of uniform strength and duration in a decelerating fashion: the resting period between two consecutive excitations grows after each pulse. Such protocols serve as a rather strict point of comparison as they have been shown to outperform LEAP analogues at lower overall PC in simulations of homogeneous 2D cardiac tissue (Lilienkamp et al., 2022b).

**FIGURE 5:**
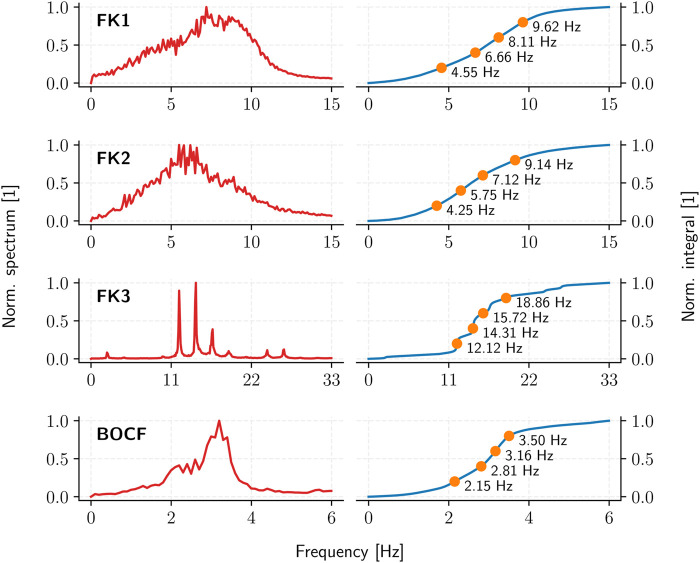
The average (squared, absolute-value) Fourier spectrum (red) and the resulting ADP inter-pulse frequencies (orange) as derived from the spectrum integral (blue) for each cardiac-tissue model (rows).

The pulse timings are derived through the Fourier spectrum of a given cardiac-tissue model: a sufficiently long (10 s) time-series of appropriate temporal resolution (10 ms) is first used to establish the spectrum and the cumulative integral of its squared absolute-value up to a given cutoff frequency. Partitioning this (normalised) integral equally according to the desired number of pulses then yields the corresponding inter-pulse periods (Figure 5).

**FIGURE 6:**
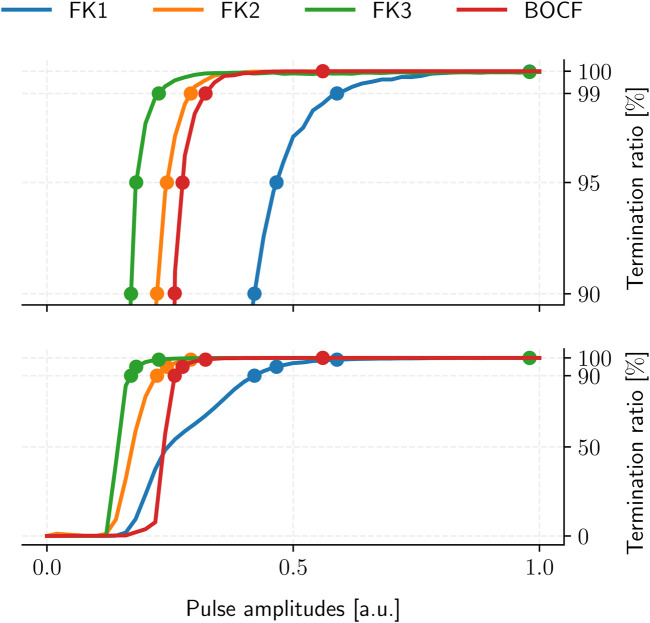
ADP protocol dose-response curves for our cardiac-tissue models (bottom) with a separate detailed view of the high-TR region (top). Dots mark each model’s PC thresholds at TR values of 90%, 95%, 99%, and 100%, respectively. Each curve was sampled at 101 equidistant amplitude values with 10^5^ termination attempts across 10 initial conditions.

The resulting ADP protocol retains one degree of freedom in its uniform pulse-amplitudes. We thus evaluated the TR of each model’s ADP protocol over multiple amplitude values to establish the corresponding PC thresholds for 90%, 95%, 99%, and 100% TR in a dose-response curve (Figure 6), yielding four reference protocols per model (Table 4).

**TABLE 4:** ADP reference protocols. Each model has one sequence of pulse periods in-between consecutive pulses as derived from its Fourier spectrum. For each model, the protocols’ uniform amplitudes were determined according to four pre-defined TR thresholds by consulting their respective dose-response curves (Figure 6).

Model	Cutoff [Hz]	Inter-p. periods [ms]				TR [%]	PC [a.u.]
FK1	15	104	123	150	220	90	1.78
						95	2.18
						99	3.46
						100	9.60
FK2	15	109	140	174	235	90	0.50
						95	0.60
						99	0.86
						100	3.14
FK3	33	53	64	70	82	90	0.30
						95	0.32
						99	0.52
						100	9.60
BOCF	6	285	316	356	466	90	0.68
						95	0.76
						99	1.04
						100	3.14

## 4 Designing a genetic algorithm

The restriction to 5-pulse pacing protocols left us with 9 degrees of freedom (5 pulse amplitudes and 4 intervals) for optimisation. We chose to use a genetic algorithm (GA) to circumvent the potential workload a brute-force approach would have entailed, which meant we had to agree on a suitable implementation for our optimisation purposes.

### 4.1 General structure

GAs attempt to emulate the evolutionary process in nature to improve upon known solutions to a problem with a measurable performance metric. While there is no definitive canonical GA, the most general approach works as follows (Eiben and Smith, 2015):

1. Take members from the current “population” of known solutions
2. Apply operators like mutation and/or crossover to generate new potential solutions
3. Assimilate the new solutions into the population

In our case, “solution” refers to a concrete pacing protocol. Each consecutive iteration of this scheme is called a *generation*.

What exactly each step entails is up to a given implementation and usually tailored to a specific problem. Despite this freedom, our particular implementation had to ensure an appropriate degree of population diversity and *ergodicity* (i.e., the algorithm’s ability to access a significant fraction of the optimisation landscape) throughout the generations: An overemphasis on population diversity would have yielded what is basically a directionless random search, and neglecting it entirely would have promoted strict elitism by favouring a restrictively narrow class of solutions instead. Either of these two cases would have compromised our GA’s efficacy in finding better pacing protocols.

The price for using a heuristic lies in the abundance of parameters introduced; these are called *hyperparameters* to distinguish them from those pertaining to the underlying optimisation problem (i.e., the pacing protocols in the context of this study) itself. GA performance is closely tied to proper hyperparameter fine-tuning, which can be an arduous process depending on the hyperparameter count of a given GA. What follows in the next few subsections are the details of our (“the”) GA, including its hyperparameters and our attempt to account for their potential influence on GA performance.

### 4.2 Mutation and crossover

**FIGURE 7:**
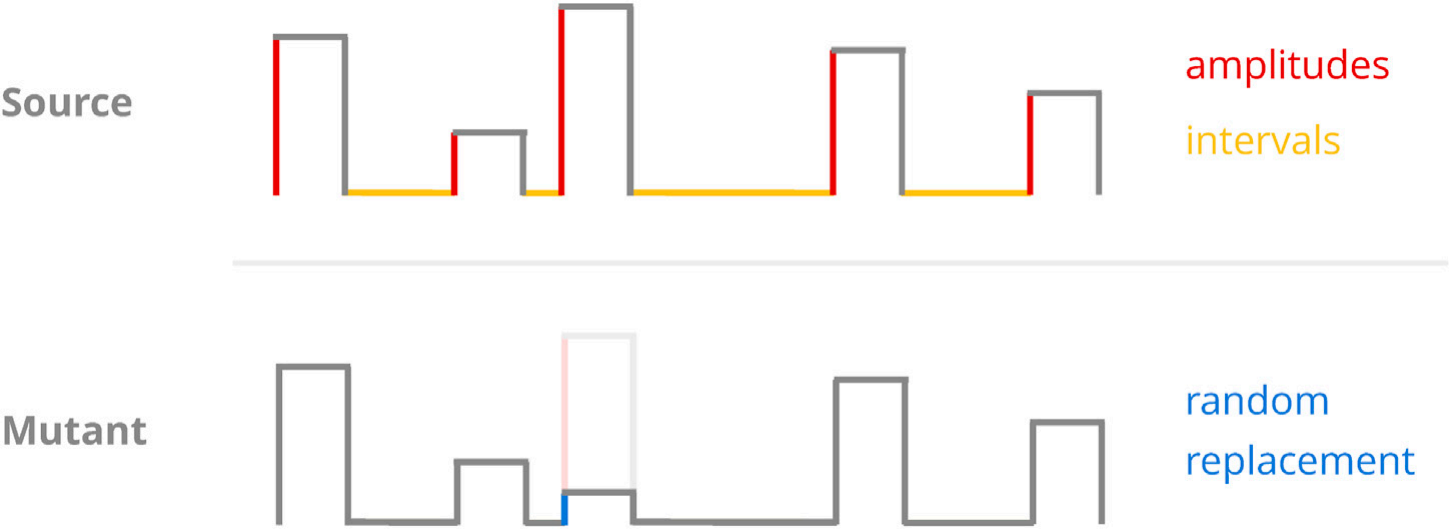
Sketch of the mutation operator. A random protocol parameter (top) is selected and replaced with a randomly chosen value from a pre-defined interval (bottom). As we only considered protocols of constant pulse length, the GA implementations only ever mutated either amplitudes or recovery intervals.

In a given generation of the GA, there is a fixed chance (defined by the *probability of mutation* hyperparameter) that a *mutation operator* will be consulted to generate a new protocol. When this happens, the protocol to be mutated is drawn randomly from the population and receives exactly one change in one of its parameters (Figure 7). The change in question is a replacement operation where the parameter’s value is replaced by one drawn from a pre-defined interval in a uniform fashion. Each type of protocol parameter (e.g., amplitudes or recovery intervals) has its own *mutation interval*, and they all constitute hyperparameters as well.

This particular mutation operator may introduce drastic (in a relative sense) change to a given parameter as it employs replacement instead of scaling. However, implementing it in this way stood to ensure a consistent driving force for ergodicity and should—in theory—have allowed for the traversal of local optima at any point during the population’s “evolution.” This mutation operator retains full access to the entire optimisation space no matter the state of the population, which may not always be the case for scaling approaches.

**FIGURE 8:**
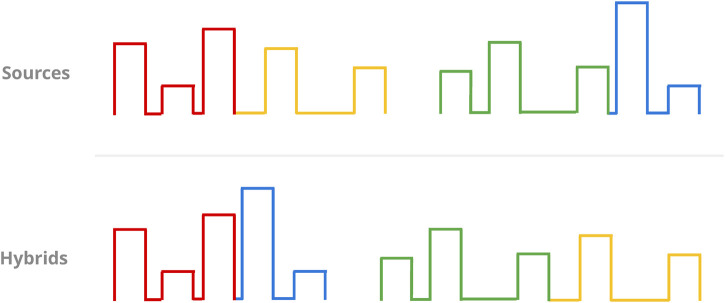
Sketch of the crossover operator. Given two randomly chosen source protocols (top, columns), a random pulse (the third one in this case) beyond the first is chosen as the cutoff point for the mergers of the resulting partial protocols (colour-coded). This results in two new protocols for selection (bottom).

Should random chance decide against mutation as a means to generating a new protocol, the GA defaults to a *crossover operator* instead. This operator generates two new protocols instead of one: The crossing of two random protocols begins with the selection of a random pulse number, excluding the final pulse to guarantee the results will not be two identical copies of the input protocols. Then, the two “parents” are split and recombined into two new protocols at the cutoff point defined by said pulse number (Figure 8).

“Parent” protocols are always drawn in a random, uniform fashion from the current population without replacement. This uniform approach should imply that no selection pressure is present in the choice of source protocols itself. Less selectivity in this context usually means greater ergodicity at the potential cost of convergence speed, but we could justify this choice with the high-dimensional nature of the protocol-optimisation landscape (and thus inherent difficulty in its traversal) at hand.

### 4.3 Selection mechanism

The GA uses a percentile-based selection process to decide whether a given protocol is to be introduced to the population or discarded: By comparing the performance metrics of the candidate (i.e., its PC and TR values) to those of the existing population, it first establishes the would-be percentile rankings for both. If and only if both rankings are sufficiently high, the protocol then passes selection. The two *acceptance percentile thresholds* to be cleared are both hyperparameters.

An accepted protocol replaces an existing one in the population, keeping the *population size* (another hyperparameter) constant. The GA determines the candidate for replacement in a two-stage process: First, it sorts the population by one metric (PC or TR) and then by the other. This establishes a ranking where the first metric of choice serves as the principal determinant of overall protocol *fitness*, while the second one is effectively a tie-breaker. The protocol at the bottom of this ranking is consequently replaced by the accepted protocol.

This assimilation scheme is biased by design, as it implicitly weighs the metric chosen for the first sorting more than the other. This ensures that even protocols excelling in one particular metric (potentially at the cost of the other) may still be subject to timely replacement. This is desirable behaviour as the selection process is percentile-based and accounts for both metrics, meaning outliers of this kind could reasonably skew the optimisation process. Avoiding this scenario should therefore counteract premature convergence. By alternating the order of metrics every time it consults such a ranking for replacement, the GA can apply this bias to either metric more or less equally.

## 5 Results

With a heuristic and reference protocols in hand, we ran computer simulations to study the GA’s viability for uncovering protocol-performance optimisation potential across our four cardiac-tissue models of choice.

### 5.1 Improvements over ADP protocols

**FIGURE 9:**
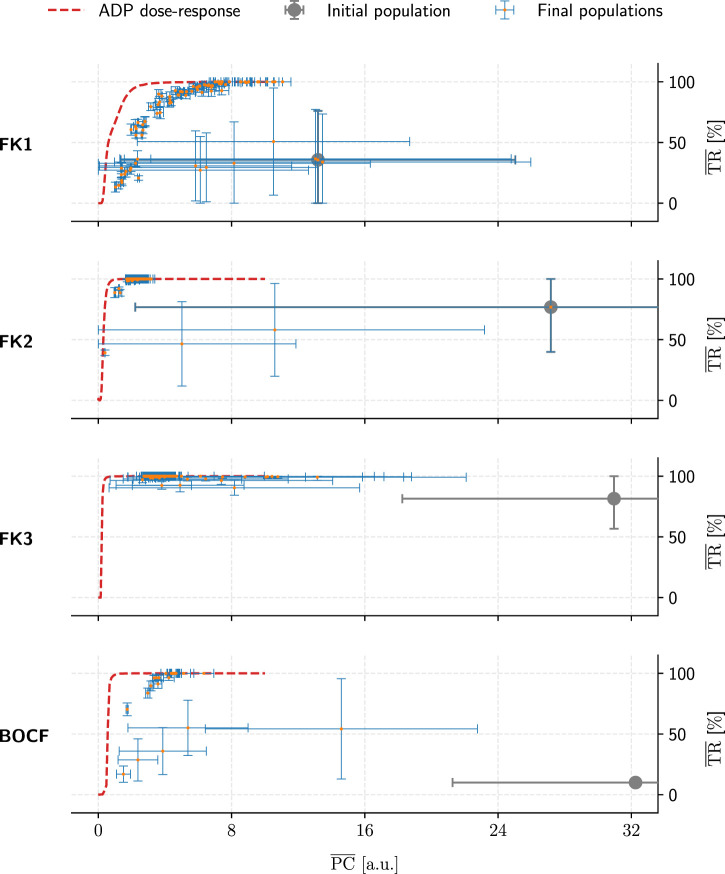
Overview of the GA-optimised populations for a single initial population (grey marker) per model (rows). These populations (comprising 50 protocols each) only differed in their choices of GA hyperparameters and are compared by their mean PC and TR values. Error bars indicate each metric’s standard deviation. Lastly, dashed red lines show the performance of ADP protocols at a given PC value.

Across all four models, our top GA-optimised protocols outperformed their ADP equivalents in terms of PC efficiency only at TR values of 99% and above (Figure 9) with some GA hyperparameter sets. At 100% TR, peak GA-mediated performance-gains across all hyperparameter sets were highest (Table 5), with, e.g., the FK3 ADP-protocol permitting a *∼*80% reduction in PC through its top GA counterpart. Comparatively, the BOCF model only saw improvements of *∼*24% over its ADP reference at peak efficacy.

**TABLE 5:** GA-mediated improvements over ADP and initial-population (pop.) protocols. Rows of protocols outperformed (or matched) by GA-optimised solutions in both metrics are highlighted in grey.

Model	Pop.	Protocol	PC [a.u.]	ΔGA [%]	TR [%]	ΔGA [p. p.]
FK1	1	Best of pop. GA	3.32		100	
		Best of init. pop.	16.90	−80	100	+0
		ADP @ 90% TR	1.78	+87	90	+10
		@ 95%	2.18	+52	95	+5
		@ 99%	3.46	−4	99	+1
		@ 100%	9.60	−65	100	+0
	2		3.00		100	
			14.40	−79	100	+0
			1.78	+69	90	+10
			2.18	+38	95	+5
			3.46	−13	99	+1
			9.60	−69	100	+0
FK2	1	Best of pop. GA	1.02		100	
		Best of init. pop.	3.60	−72	100	+0
		ADP @ 90% TR	0.50	+104	90	+10
		@ 95%	0.60	+70	95	+5
		@ 99%	0.86	+19	99	+1
		@ 100%	3.14	−68	100	+0
	2		0.98		100	
			3.60	−73	100	+0
			0.50	+96	90	+10
			0.60	+63	95	+5
			0.86	+14	99	+1
			3.14	−69	100	+0
FK3	1	Best of pop. GA	1.90		100	
		Best of init. pop.	8.82	−78	100	+0
		ADP @ 90% TR	0.30	+533	90	+10
		@ 95%	0.32	+494	95	+5
		@ 99%	0.52	+265	99	+1
		@ 100%	9.60	−80	100	+0
	2		1.88		100	
			9.38	−80	100	+0
			0.30	+527	90	+10
			0.32	+488	95	+5
			0.52	+262	99	+1
			9.60	−80	100	+0
BOCF	1	Best of pop. GA	2.40		100	
		Best of init. pop.	3.82	−37	10	+90
		ADP @ 90% TR	0.68	+253	90	+10
		@ 95%	0.76	+216	95	+5
		@ 99%	1.04	+131	99	+1
		@ 100%	3.14	−24	100	+0
	2		3.00		100	
			15.02	−80	100	+0
			0.68	+341	90	+10
			0.76	+295	95	+5
			1.04	+188	99	+1
			3.14	−4	100	+0

Likely due to excessive selection pressure maximising the termination rate, most GA populations approached a PC-TR saturation curve that falls beneath the respective ADP dose-response, implying overall worse PC efficiency at comparable TR. The GA’s “catching up” to overall ADP-protocol performance at higher TR values may have been owed to the asymptotic nature of the ADP dose-response curves (Figure 6): There, a TR approaching 100% demands disproportionally higher PC, enlarging the optimisation space for discovering potentially more efficient alternatives by means of GA optimisation.

In contrast, the GA achieved consistent performance gains (up to *∼*80% PC reductions at peak TR) within the initial populations for each model. This indicates that the overall heuristic does, in fact, operate as expected in optimising a given protocol population toward more efficient pacing schemes.

### 5.2 Population-convergence behaviour and robustness

**FIGURE 10:**
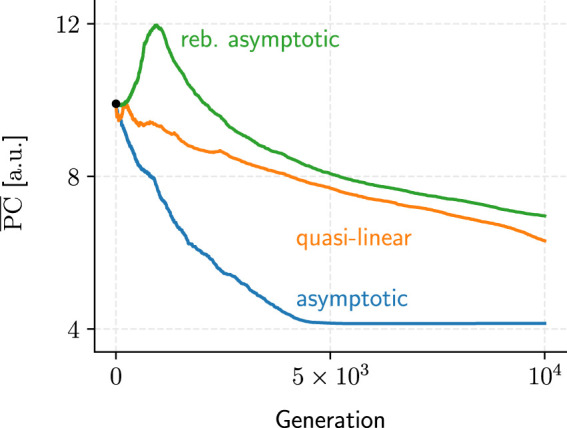
Samples for each of the three recurring metric-performance patterns we observed over the course of the GA evaluations. Only PC examples are shown because TR convergence generally exhibited similar patterns (albeit mirrored horizontally).

We identified three recurring convergence patterns in the populations’ mean performance metrics, PC and TR, over the hundreds of GA evaluations conducted (Figure 10): *asymptotic*, *rebounding asymptotic*, and *quasi-linear* convergence. While asymptotic convergence was the most common, its rebounding variant could be seen as evidence that the GA is not a fully “greedy” algorithm and should be capable of navigating local optima to some extent.

**FIGURE 11:**
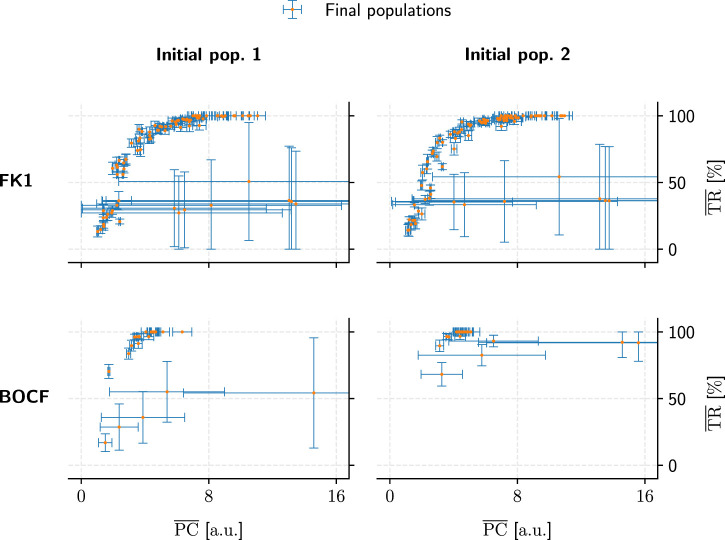
Comparison of population convergence under different initial populations (columns) as well as models (rows). Each final population consisted of 50 pacing protocols and is placed according to its mean PC and TR values. Error bars indicate the corresponding standard deviations.

Metric convergence behaviour was stable under different initial populations (Figure 11) for all models. A direct link between hyperparameters and metric convergence is difficult to establish, however, since our two population-initialisation schemes likely influenced the outcome to some degree. As we only employed one of these initialisation methods for a given model, we lack the means to compare the two and adequately quantify this potential bias after the fact.

The substantial PC and TR standard-deviations for some populations in Figures 9, 11 show that not every hyperparameter combination managed to fully converge within the allotted 10^4^ generations. Fully converged data points cluster chiefly along an implied saturation curve along the TR axis where the lower end of PC values lie; while this could be an indicator that the GA is principally operating in the correct direction of the PC-TR optimisation plane, the question remains whether these partially converged GA setups would have yielded superior results than those of their more rapidly converging counterparts eventually.

### 5.3 Emerging protocol patterns

**FIGURE 12:**
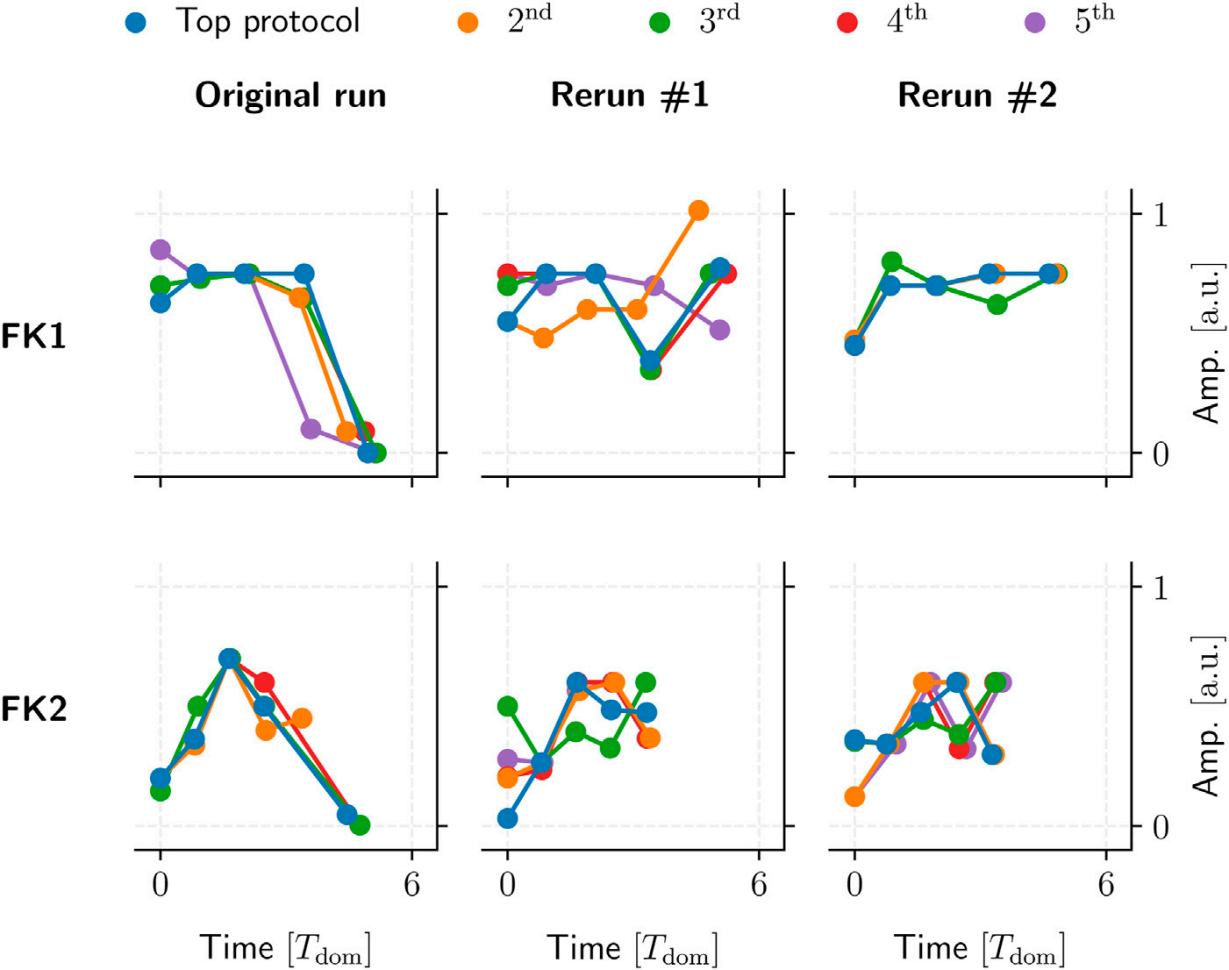
Five examples of top GA-optimised protocols for two FK models (rows). These protocols had the highest TR with the lowest PC among their respective populations. Said populations were generated by a single 10^4^-generation application of our GA using one set of hyperparameters and initial population per model.

We could not identify any overarching, reproducible protocol patterns among the GA-optimised top performers. Even reruns using the same initial population and GA hyperparameters would yield different protocol structures due to statistical variance alone (Figure 12).

Our GA-mediated protocols likely fall into one of potentially many fine-grained local minima, past any generalisable structures of note. This could also tie in with the GA’s overall incentive to optimise toward a (mathematically strictly optimal) TR of 100%, which may overemphasise overly specific protocols to the possible detriment of generalisable, similarly high-performing (e.g., *∼*90% TR) patterns.

## 6 Summary and discussion

With a grasp of the GA’s overall performance and optimisation efficacy, we can now put our results in the larger context of our goal of protocol-performance optimisation and identify potential avenues for improvement and future research into this topic.

We successfully established pacing-protocol optimisation potential between *∼*4% and *∼*80% over our similarly effective (in terms of TR) ADP reference protocols (Table 5). While we have managed to improve on our GA’s initial populations on the order of *∼*80% overall PC reduction at peak TR, the overall lacklustre comparison to otherwise high-performing ADP protocols (Figure 9) shows room for improvement in the general optimisation heuristic. There may also be generalisable protocol patterns to be discovered within the realm of close-to-peak-efficacy (*∼*90% and above) protocols as a whole.

The main insight from our findings thus concerns the idea that optimisation toward a 100% success rate may be overly restrictive as far as protocol-pattern discoverability is concerned, and an equivalence (in terms of protocol fitness) among TRs above *∼*90% may present a worthwhile avenue of investigation in the future.

The GA implementation was based on mutation and crossover operators with percentile-based selection for the assimilation of new protocols into a given population. It proved consistently capable of significantly improving all models’ initial protocol-populations, but the lack of recurrently emerging protocol patterns leaves us without concrete insight into the general properties and patterns of an efficient pacing protocol.

We used only four cardiac-tissue models: Three FK and one BOCF, along with arbitrarily selected parameter sets. Each model was considered in isolation, while cross-optimisation could perhaps have helped guide the optimisation toward more general protocol structures. While we managed to replicate some of the GA’s behaviour across these individual models, this was done using simplified two-dimensional simulations of fibrillation on square sheets of tissue only. Naturally, more realistic geometries along with physiological cardiac-tissue models might yield results more immediately applicable to traditional experimental settings.

Our pacing model was based on homogeneous cardiac tissue with randomly distributed, square-shaped injection sites serving as an abstraction of local tissue heterogeneities responsible for emitting wave fronts when exposed to potent electric fields as utilised by LEAP. The number of sites, their sizes, and their shape(s) were all set to rather arbitrary values for simplicity’s sake, leaving yet another potential avenue for further investigation as to their effect(s) on GA performance. Perhaps more sophisticated site-distribution algorithms beyond random placement could help mimic real heterogeneity distributions more closely in this context, but the indirect implementation of virtual electrodes through the simulation of actual heterogeneous tissue domains may also be worth considering.

Lastly, the particular GA setup we employed is merely one of many possible alternatives, and others may prove yet more effective at optimising pacing protocols. We used *either* mutation or crossover to generate one or two new protocols in a given generation and selected the respective source protocol(s) entirely randomly every time, which deviates from the more “canonical” approach (phrased in the context of pacing protocols) of using, e.g., tournament selection (Blickle and Thiele, 1996) to evolve the protocol population.

The next step in computer-aided pacing optimisation could entail the cross-optimisation with multiple models, fitness equivalence of TR values beyond *∼*90%, transition to three-dimensional simulations, more sophisticated pacing models, the testing of alternative GA implementations, or a combination thereof. We established a foothold in pacing optimisation through the outline of this paper, but more work into studying the GA, its behaviour, and its shortcomings is needed before considerable time and effort is spent working with, e.g., physiological models in more complex geometries beyond square sheets of cardiac tissue.

Other popular heuristics like *simulated annealing* (Ingber, 1993) may also warrant investigation for their efficacy in protocol optimisation based on our pacing model. It would at the very least be pertinent to compare their results to our GA’s, especially in light of the convergence issues we have uncovered over the course of the GA-based optimisation effort.

Further optimisation of anti-fibrillation control may be achieved by proper timing of the application of the pulse sequence, as it has recently been shown that the timing of single-shock termination has a significant influence on the probability of success (Ji et al., 2017; Steyer et al., 2023).

We can conclude this paper with a successful foray into pacing-protocol optimisation where we showcased the optimisation capabilities of a simple genetic algorithm for randomised protocol populations, as well as substantial pacing-energy reductions over ADP protocols at 100% sucess rate. In the process, we identified multiple avenues of investigation for future projects; said projects could focus on general improvements to the GA design for better protocols or taking the simulations to a more realistic level to better facilitate their translation into experiments on live cardiac tissue for verification purposes.

## Data Availability

The raw data supporting the conclusion of this article will be made available by the authors, without undue reservation.

## References

[B1] Aron M. Herzog S. Parlitz U. Luther S. Lilienkamp T. (2019). Spontaneous termination of chaotic spiral wave dynamics in human cardiac ion channel models. PLOS ONE 14, e0221401–e0221416. 10.1371/journal.pone.0221401 31461472 PMC6713330

[B2] Babbs C. F. Tacker W. A. VanVleet J. F. Bourland J. D. Geddes L. A. (1980). Therapeutic indices for transchest defibrillator shocks: Effective, damaging, and lethal electrical doses. Am. Heart J. 99, 734–738. 10.1016/0002-8703(80)90623-7 7377095

[B3] Bittihn P. Hörning M. Luther S. (2012). Negative curvature boundaries as wave emitting sites for the control of biological excitable media. Phys. Rev. Lett. 109, 118106. 10.1103/PhysRevLett.109.118106 23005683

[B4] Bittihn P. Luther G. Bodenschatz E. Krinsky V. Parlitz U. Luther S. (2008). Far field pacing supersedes anti-tachycardia pacing in a generic model of excitable media. New J. Phys. 10, 103012. 10.1088/1367-2630/10/10/103012 PMC2944386 20368243

[B5] Bittihn P. Squires A. Luther G. Bodenschatz E. Krinsky V. Parlitz U. (2010). Phase-resolved analysis of the susceptibility of pinned spiral waves to far-field pacing in a two-dimensional model of excitable media. Phil. Trans. R. Soc. A 368, 2221–2236. 10.1098/rsta.2010.0038 20368243 PMC2944386

[B6] Blickle T. Thiele L. (1996). A comparison of selection schemes used in evolutionary algorithms. Evol. Comput. 4, 361–394. 10.1162/evco.1996.4.4.361

[B7] Boccia E. Luther S. Parlitz U. (2017a). Modelling far field pacing for terminating spiral waves pinned to ischaemic heterogeneities in cardiac tissue. Phil. Trans. R. Soc. A 375, 20160289. 10.1098/rsta.2016.0289 28507234 PMC5434080

[B8] Boccia E. Parlitz U. Luther S. (2017b). Spontaneous termination of reentrant activity under myocardial acute ischemia: Role of cellular conductivity and its relation to ischemic heterogeneities. Commun. Nonlinear Sci. Numer. Simul. 48, 115–122. 10.1016/j.cnsns.2016.12.014

[B9] Bruegmann T. Boyle P. M. Vogt C. C. Karathanos T. V. Arevalo H. J. Fleischmann B. K. (2016). Optogenetic defibrillation terminates ventricular arrhythmia in mouse hearts and human simulations. J. Clin. Investigation 126, 3894–3904. 10.1172/JCI88950 PMC5096832 27617859

[B10] Bueno-Orovio A. Cherry E. M. Fenton F. H. (2008). Minimal model for human ventricular action potentials in tissue. J. Theor. Biol. 253, 544–560. 10.1016/j.jtbi.2008.03.029 18495166

[B11] Buran P. Bär M. Alonso S. Niedermayer T. (2017). Control of electrical turbulence by periodic excitation of cardiac tissue. Chaos An Interdiscip. J. Nonlinear Sci. 27, 113110. 10.1063/1.5010787 29195336

[B12] Buran P. Niedermayer T. Bär M. (2023). Mechanism of defibrillation of cardiac tissue by periodic low-energy pacing. Available at:https://www.biorxiv.org/content/10.1101/2023.03.16.533010v1 (Accessed March 20, 2023).

[B13] Buran P. Niedermayer T. Bär M. (2022). Suppression of fibrillatory dynamics consisting of stable rotors by periodic pacing. New J. Phys. 24, 083024. 10.1088/1367-2630/ac8571

[B14] Cherry E. M. Fenton F. H. (2008). Visualization of spiral and scroll waves in simulated and experimental cardiac tissue. New J. Phys. 10, 125016. 10.1088/1367-2630/10/12/125016

[B15] Christoph J. Chebbok M. Richter C. Schröder-Schetelig J. Bittihn P. Stein S. (2018). Electromechanical vortex filaments during cardiac fibrillation. Nature 555, 667–672. 10.1038/nature26001 29466325

[B16] Davidenko J. M. Pertsov A. V. Salomonsz R. Baxter W. Jalife J. (1992). Stationary and drifting spiral waves of excitation in isolated cardiac muscle. Nature 355, 349–351. 10.1038/355349a0 1731248

[B17] DeTal N. Fenton F. (2021). Optimizing low-energy anti-fibrillation pacing: Lessons from a cellular automaton model. Available at: https://arxiv.org/abs/2109.10861 (Accessed September 22, 2021).

[B18] DeTal N. Kaboudian A. Fenton F. H. (2022). Terminating spiral waves with a single designed stimulus: Teleportation as the mechanism for defibrillation. Proc. Natl. Acad. Sci. 119, e2117568119. 10.1073/pnas.2117568119 35679346 PMC9214532

[B19] Efimov I. Ripplinger C. M. (2006). Virtual electrode hypothesis of defibrillation. Heart rhythm. 3, 1100–1102. 10.1016/j.hrthm.2006.03.005 16945810

[B20] Eiben A. Smith J. (2015). “Introduction to evolutionary computing,” in Natural computing series. 2 (Berlin Heidelberg: Springer-Verlag).

[B21] Fenton F. Cherry E. Hastings H. Evans S. (2002). Multiple mechanisms of spiral wave breakup in a model of cardiac electrical activity. Chaos An Interdiscip. J. Nonlinear Sci. 12, 852–892. 10.1063/1.1504242 12779613

[B22] Fenton F. H. Luther S. Cherry E. M. Otani N. F. Krinsky V. Pumir A. (2009). Termination of atrial fibrillation using pulsed low-energy far-field stimulation. Circulation 120, 467–476. 10.1161/CIRCULATIONAHA.108.825091 19635972 PMC2867100

[B23] Fenton F. Karma A. (1998). Vortex dynamics in three-dimensional continuous myocardium with fiber rotation: Filament instability and fibrillation. Chaos An Interdiscip. J. Nonlinear Sci. 8, 20–47. 10.1063/1.166311 12779708

[B24] Godemann F. Butter C. Lampe F. Linden M. Schlegl M. Schultheiss H.-P. (2004). Panic disorders and agoraphobia: Side effects of treatment with an implantable cardioverter/defibrillator. Clin. Cardiol. 27, 321–326. 10.1002/clc.4960270604 15237689 PMC6654734

[B25] Gray R. A. Pertsov A. M. Jalife J. (1998). Spatial and temporal organization during cardiac fibrillation. Nature 392, 75–78. 10.1038/32164 9510249

[B26] Hornung D. Biktashev V. N. Otani N. F. Shajahan T. K. Baig T. Berg S. (2017). Mechanisms of vortices termination in the cardiac muscle. R. Soc. Open Sci. 4, 170024. 10.1098/rsos.170024 28405398 PMC5383855

[B27] Hussaini S. Venkatesan V. Biasci V. Romero Sepúlveda J. M. Quiñonez Uribe R. A. Sacconi L. (2021). Drift and termination of spiral waves in optogenetically modified cardiac tissue at sub-threshold illumination. eLife 10, e59954. 10.7554/eLife.59954 33502313 PMC7840178

[B28] Ingber L. (1993). Simulated annealing: Practice versus theory. Math. Comput. Model. 18, 29–57. 10.1016/0895-7177(93)90204-C

[B29] Janardhan A. H. Gutbrod S. R. Li W. Lang D. Schuessler R. B. Efimov I. R. (2014). Multistage electrotherapy delivered through chronically-implanted leads terminates atrial fibrillation with lower energy than a single biphasic shock. J. Am. Coll. Cardiol. 63, 40–48. 10.1016/j.jacc.2013.07.098 24076284 PMC4123180

[B30] Ji Y. C. Uzelac I. Otani N. Luther S. Gilmour R. F. Cherry E. M. (2017). Synchronization as a mechanism for low-energy anti-fibrillation pacing. Heart rhythm. 14, 1254–1262. 10.1016/j.hrthm.2017.05.021 28502873

[B31] Li Z. Qiao Z. Tang T. (2018). Numerical solution of differential equations: Introduction to finite difference and finite element methods. 1st. Cambridge: Cambridge University Press.

[B32] Lilienkamp T. Christoph J. Parlitz U. (2017). Features of chaotic transients in excitable media governed by spiral and scroll waves. Phys. Rev. Lett. 119, 054101. 10.1103/PhysRevLett.119.054101 28949756

[B33] Lilienkamp T. Parlitz U. Luther S. (2022a). Non-monotonous dose response function of the termination of spiral wave chaos. Sci. Rep. 12, 12043. 10.1038/s41598-022-16068-8 35835979 PMC9283470

[B34] Lilienkamp T. Parlitz U. Luther S. (2022b). Taming cardiac arrhythmias: Terminating spiral wave chaos by adaptive deceleration pacing. Chaos An Interdiscip. J. Nonlinear Sci. 32, 121105. 10.1063/5.0126682 36587312

[B35] Lilienkamp T. Parlitz U. (2018). Terminal transient phase of chaotic transients. Phys. Rev. Lett. 120, 094101. 10.1103/PhysRevLett.120.094101 29547310

[B36] Lilienkamp T. Parlitz U. (2020). Terminating transient chaos in spatially extended systems. Chaos An Interdiscip. J. Nonlinear Sci. 30, 051108. 10.1063/5.0011506 32491910

[B37] Loppini A. Erhardt J. Fenton F. H. Filippi S. Hörning M. Gizzi A. (2022). Optical ultrastructure of large mammalian hearts recovers discordant alternans by *in silico* data assimilation. Front. Netw. Physiology 2, 866101. 10.3389/fnetp.2022.866101 PMC10012998 36926104

[B38] Luther S. Fenton F. H. Kornreich B. G. Squires A. Bittihn P. Hornung D. (2011). Low-energy control of electrical turbulence in the heart. Nature 475, 235–239. 10.1038/nature10216 21753855 PMC3153959

[B39] Lynch R. E. (1992). Fundamental solutions of nine-point discrete Laplacians. Appl. Numer. Math. 10, 325–334. 10.1016/0168-9274(92)90048-i

[B40] Mackenzie D. (2004). Making sense of a heart gone wild. Science 303, 786–787. 10.1126/science.303.5659.786 14764864

[B41] Marcus G. M. Chan D. W. Redberg R. F. (2011). Recollection of pain due to inappropriate versus appropriate implantable cardioverter-defibrillator shocks. cardioverter-defibrillator shocks 34, 348–353. 10.1111/j.1540-8159.2010.02971.x PMC3057533 21077915

[B42] Mehra R. (2007). Global public health problem of sudden cardiac death. J. Electrocardiol. 40, S118–S122. 10.1016/j.jelectrocard.2007.06.023 17993308

[B43] Moss A. J. Schuger C. Beck C. A. Brown M. W. Cannom D. S. Daubert J. P. (2012). Reduction in inappropriate therapy and mortality through ICD programming. N. Engl. J. Med. 367, 2275–2283. 10.1056/NEJMoa1211107 23131066

[B44] Otani N. F. Wheeler K. Krinsky V. Luther S. (2019). Termination of scroll waves by surface impacts. Phys. Rev. Lett. 123, 068102. 10.1103/PhysRevLett.123.068102 31491191 PMC7381943

[B45] Poole J. E. Johnson G. W. Hellkamp A. S. Anderson J. Callans D. J. Raitt M. H. (2008). Prognostic importance of defibrillator shocks in patients with heart failure. N. Engl. J. Med. 359, 1009–1017. 10.1056/NEJMoa071098 18768944 PMC2922510

[B46] Pumir A. Krinsky V. (1999). Unpinning of a rotating wave in cardiac muscle by an electric field. J. Theor. Biol. 199, 311–319. 10.1006/jtbi.1999.0957 10433895

[B47] Pumir A. Nikolski V. Hörning M. Isomura A. Agladze K. Yoshikawa K. (2007). Wave emission from heterogeneities opens a way to controlling chaos in the heart. Phys. Rev. Lett. 99, 208101. 10.1103/PhysRevLett.99.208101 18233188

[B48] Rantner L. J. Tice B. M. Trayanova N. A. (2013). Terminating ventricular tachyarrhythmias using far-field low-voltage stimuli: Mechanisms and delivery protocols. Heart rhythm. 10, 1209–1217. 10.1016/j.hrthm.2013.04.027 23628521 PMC3735828

[B49] Rappel W.-J. Krummen D. E. Baykaner T. Zaman J. Donsky A. Swarup V. (2022). Stochastic termination of spiral wave dynamics in cardiac tissue. Front. Netw. Physiology 2, 809532. 10.3389/fnetp.2022.809532 PMC9524168 36187938

[B50] Schwaderlapp G. Oesterlein T. Dössel O. Armin L. Schmitt C. Lenis G. (2017). Definition, estimation and limitations of the dominant frequency in intracardiac electrograms. Curr. Dir. Biomed. Eng. 3, 95–98. 10.1515/cdbme-2017-0175

[B51] Shajahan T. K. Berg S. Luther S. Krinski V. Bittihn P. (2016). Scanning and resetting the phase of a pinned spiral wave using periodic far field pulses. New J. Phys. 18, 043012. 10.1088/1367-2630/18/4/043012

[B52] Steyer J. Lilienkamp T. Luther S. Parlitz U. (2023). The role of pulse timing in cardiac defibrillation. Front. Netw. Physiology 2, 1007585. 10.3389/fnetp.2022.1007585 PMC10013017 36926106

[B53] Strain M. C. Greenside H. S. (1998). Size-dependent transition to high-dimensional chaotic dynamics in a two-dimensional excitable medium. Phys. Rev. Lett. 80, 2306–2309. 10.1103/PhysRevLett.80.2306

[B54] Tereshchenko L. G. Faddis M. N. Fetics B. J. Zelik K. E. Efimov I. R. Berger R. D. (2009). Transient local injury current in right ventricular electrogram after implantable cardioverter-defibrillator shock predicts heart failure progression. J. Am. Coll. Cardiol. 54, 822–828. 10.1016/j.jacc.2009.06.004 19695461 PMC2803080

[B55] tom Wörden H. Parlitz U. Luther S. (2019). Simultaneous unpinning of multiple vortices in two-dimensional excitable media. Phys. Rev. E 99, 042216. 10.1103/PhysRevE.99.042216 31108599

[B56] Trayanova N. A. Chang K. C. (2016). How computer simulations of the human heart can improve anti-arrhythmia therapy. J. Physiol. 594, 2483–2502. 10.1113/JP270532 26621489 PMC4850196

[B57] Trayanova N. A. Constantino J. L. Ashihara T. Plank G. (2011). Modeling defibrillation of the heart: Approaches and insights. IEEE Rev. Biomed. Eng. 4, 89–102. 10.1109/RBME.2011.2173761 22273793 PMC3328410

[B58] Winfree A. T. (1989). Electrical instability in cardiac muscle: Phase singularities and rotors. J. Theor. Biol. 138, 353–405. 10.1016/s0022-5193(89)80200-0 2593680

[B59] Witkowski F. X. Leon L. J. Penkoske P. A. Giles W. R. Spano M. L. Ditto W. L. (1998). Spatiotemporal evolution of ventricular fibrillation. Nature 392, 78–82. 10.1038/32170 9510250

[B60] Xie J. Weil M. H. Sun S. Tang W. Sato Y. Jin X. (1997). High-energy defibrillation increases the severity of postresuscitation myocardial dysfunction. Circulation 96, 683–688. 10.1161/01.CIR.96.2.683 9244243

